# Molecular Changes in the Cardiac RyR2 With Catecholaminergic Polymorphic Ventricular Tachycardia (CPVT)

**DOI:** 10.3389/fphys.2022.830367

**Published:** 2022-02-10

**Authors:** Angela F. Dulhunty

**Affiliations:** Muscle Research Group, John Curtin School of Medical Research, Eccles Institute of Neuroscience, Australian National University, Canberra, ACT, Australia

**Keywords:** cardiac ryanodine receptor, Ca^2+^ signaling, catecholaminergic ventricular tachycardia, cardiac arrhythmia, Ca^2+^ release channel

## Abstract

The cardiac ryanodine receptor Ca^2+^ release channel (RyR2) is inserted into the membrane of intracellular sarcoplasmic reticulum (SR) myocyte Ca^2+^ stores, where it releases the Ca^2+^ essential for contraction. Mutations in proteins involved in Ca^2+^ signaling can lead to catecholaminergic polymorphic ventricular tachycardia (CPVT). The most common cellular phenotype in CPVT is higher than normal cytoplasmic Ca^2+^ concentrations during diastole due to Ca^2+^ leak from the SR through mutant RyR2. Arrhythmias are triggered when the surface membrane sodium calcium exchanger (NCX) lowers cytoplasmic Ca^2+^ by importing 3 Na^+^ ions to extrude one Ca^2+^ ion. The Na^+^ influx leads to delayed after depolarizations (DADs) which trigger arrhythmia when reaching action potential threshold. Present therapies use drugs developed for different purposes that serendipitously reduce RyR2 Ca^2+^ leak, but can adversely effect systolic Ca^2+^ release and other target processes. Ideal drugs would specifically reverse the effect of individual mutations, without altering normal channel function. Such drugs will depend on the location of the mutation in the 4967-residue monomer and the effect of the mutation on local structure, and downstream effects on structures along the conformational pathway to the pore. Such atomic resolution information is only now becoming available. This perspective provides a summary of known or predicted structural changes associated with a handful of CPVT mutations. Known molecular changes associated with RyR opening are discussed, as well one study where minute molecular changes with a particular mutation have been tracked from the N-terminal mutation site to gating residues in the channel pore.

## Introduction

A group of inherited arrhythmogenic conditions fall under the CPVT umbrella. In these conditions the heart is structurally normal, but patients are susceptible to catecholamine-mediated ventricular arrhythmias, syncope, bradycardia and sudden death with exercise or stress, as well as sudden death during sleep (Olubando et al., 2020). CPVT can be caused by mutations any one of the proteins contributing to cardiac excitation-contraction (EC) coupling and have been grouped into eight variants (Priori et al., 2021). Two variants characterized by diastolic Ca^2+^ leak inducing DADs are CPVT-1 caused by gain-of-function mutations in RyR2, and CPVT-2 caused by mutations in calsequestrin (CSQ2), the Ca^2+^ binding protein within the cardiac SR. Six additional variants are typified by DADs or early-afterdepolarizations (EADs) associated with mutations in calmodulin and triadin isoforms, and *Trans*-2,3-enoyl-CoA reductase-like protein (TECRL). These variants include loss-of-function RyR2 mutations, where arrhythmia is not catecholaminergic, depending instead on electrical remodeling leading to arrhythmia through EAD re-entry (Zhong et al., 2021). Clearly, very different strategies will be required to treat arrhythmia arising from mutations in different proteins and also RyR2 mutations associated with gain-of-function or loss-of-function. The emphasis in this perspective is on the >170 gain-of-function RyR2 mutations that lead to CPVT-1 (Priori et al., 2021).

## The Distribution of Catecholaminergic Polymorphic Ventricular Tachycardia Mutations

A recent study of 326 RyR2 mutations including control (non-pathogenic) and CPVT-associated groups found that many of the mutations were located in four CPVT hot spot regions: 10.7% in region I (residues 77–466); 14.7% in region II (2246–2534); 21.5% in region III (3778–4201) and 21.2% in region IV (4497–4949), while 31.9% were situated outside hotspot regions (Olubando et al., 2020). More CPVT-associated mutations than control were located between central domain residues 3949–4332 and between transmembrane domain residues 4867–4967. On the other hand, more control than CPVT variants were located in the three SPRY domains (residues 652–1641) and helical domain HD2 (residues 2906–3826). The 47 sudden death mutations were scattered amongst other CPVT mutations, while the sudden death during sleep group were in mainly in the transmembrane and C-terminal domain (residues 4333–4967).

**FIGURE 1:**
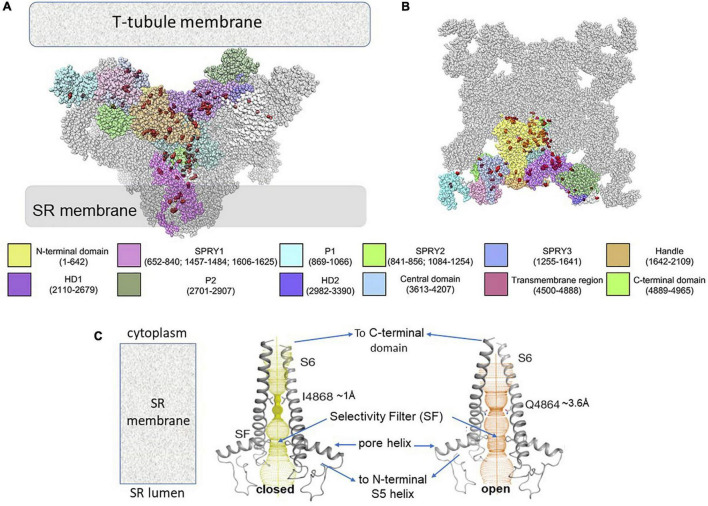
Distribution on RyR2 structure of gain-of-function RyR2 CPVT mutations and changes in RyR2 pore with channel opening, likely to characterize the pore with CPVT. **(A)** A side view of RyR2 as viewed in its location between the transverse (T) – tubule and SR membranes. Three of the four RyR monomers forming the tetrameric channel are shown, with the various domains of the central monomer are color coded. The fourth monomer is hidden behind the three monomers. **(B)** A cytosolic section through the tetramer perpendicular to the view in **(A)** and parallel to the T-tubule membrane. In **(A,B)**, 108 human gain-of-function mutations (Priori et al., 2021) are shown as red spheres. Color codes for domains in (a) and (b) are given along with residue numbers. HD – helical domain. The figure has been modified from Figure 4 (Priori et al., 2021) under copyright License #5194490720614. Labeling only has been modified. **(C)** The pore is shown traversing the SR membrane. In the closed conformation (left) the pore is constricted to ∼1 Å at I4868. In the open conformation the constriction expands to ∼3.6 Å with the minimum at Q4864. There is a second constriction at the selectivity filter (SF) near the junction of the pore helix and S6 helix which is similar in the open and closed state. On the luminal side of the pore the N-terminus of S6 is continuous with the pore helix and then the S5 helix which connects to the reminder of the transmembrane domain and the cytoplasmic central domain. The cytoplasmic C-terminal end of S6 is continuous with the C-terminal domain which shares interdomain interactions with central domain as indicated in the figure. The Figure has been modified from Figure 3 (Gong et al., 2019) under copyright License # 5194601053471. Labeling only has been modified.

Human gain-of-function CPVT-1 mutations extend from the extreme cytoplasmic surface of RyR2 facing the T-tubule membrane through to deeper parts of the protein (Figures 1A,B; Priori et al., 2021), along intra- and inter-subunit interfaces to the central and C-terminal domains into the SR transmembrane domain and channel pore. In contrast, loss-of-function RyR2 mutations are located mainly in transmembrane and C-terminal domains (Zhong et al., 2021).

## Broad Mechanistic Models of “Leaky” RyRs

Data emerging from high resolution cryo-EM is now revealing the structural impact of CPVT mutations at a near-atomic level, as described below. However, the functional significance of the mutations has been understood for some time in terms of three generalized models for gain-of-function mutations fully reviewed by Priori et al. (2021) and summarized below.

The “unzipping” model suggests that low resting RyR1 and RyR2 activity depends on interactions between the N-terminal and central domains of the protein that favor the closed state (Shtifman et al., 2002; Kobayashi et al., 2009; Suetomi et al., 2011). Interruption of the interactions by mutations, causes “unzipping”, which increases channel open probability, while drugs (e.g., dantrolene) that restore domain zipping reduce open probability and “leak”.

The store overload induced Ca^2+^ release (SOICR) model, depends on the observation that RyR2 open probability increases when the SR luminal Ca^2+^ reaches a critical threshold concentration as the SR refills during diastole (Jiang et al., 2008). Gain-of-function RyR2 mutations lower this threshold, so that the channel opens with less SR Ca^2+^ accumulation.

The RyR2 phosphorylation/FKBP12.6 model suggests that the 12.6 Kda FK506 binding protein FKBP12.6 (aka calstabin2) stabilizes RyR2 closure during diastole and its binding to RyR2 is disrupted by RyR2 phosphorylation following adrenergic stimulation (Wehrens et al., 2003; Connell et al., 2020).

The models are not mutually exclusive since FKBP12.6 dissociation could lead to unzipping and unzipping could reduce SOICR threshold. The models are sufficiently general to be adjusted molecular changes as they become available for individual mutations. However, without residue specific conformational information the models alone will not underpin the development of mutation-specific drugs.

## High Resolution Structural Analysis of Catecholaminergic Polymorphic Ventricular Tachycardia Mutations

The development of high resolution cryo-EM has revolutionized our understanding of structural changes in the RyR protein associated with channel gating. A general description of WT RyR2 channel gating is outlined first before considering the effects of CPVT mutations. Starting at the transmembrane domain, the RyR2 channel pore is formed by the four S6 inner helices. The “gate” in the closed state at I4868 constricts the ion pathway to a ∼1 A*^o^* diameter (Figure 1C; Peng et al., 2016; Gong et al., 2019). In the open channel, the minimum pore diameter of ∼4 A*^o^* at Q4864 allows ion passage. The S6 helices move to open the channel in response to conformational changes the C-terminal domain (Figure 1C). The four C-terminal domains form a flexible ring around the pore that is stabilized by electrostatic interactions between C-terminal residues on neighboring subunits. The open transduction occurs when these interactions become weaker and the ring expands to realign the S6 helices and open the pore. Numerous disease-associated mutations are located on this critical inter-subunit interface. The C-terminal domain effectively forms a joint between the S6 helix and the central domain which transmits stearic changes from the central domain to the pore (Peng et al., 2016; Gong et al., 2019). The conformation of the central domain is influenced by structural changes the peripheral N-terminal, helical and other outer cytoplasmic domains *via* interactions between residues in opposing inter-domain interfaces. Thus, structurally connected pathways exist from the most distant regions of the RyR to the channel gate. Channel regulators (including Ca^2+^, Mg^2 +^, ATP, Calmodulin and intrinsic modulatory proteins), mutations, and drug binding at any point along this pathway, can alter gating.

**FIGURE 2:**
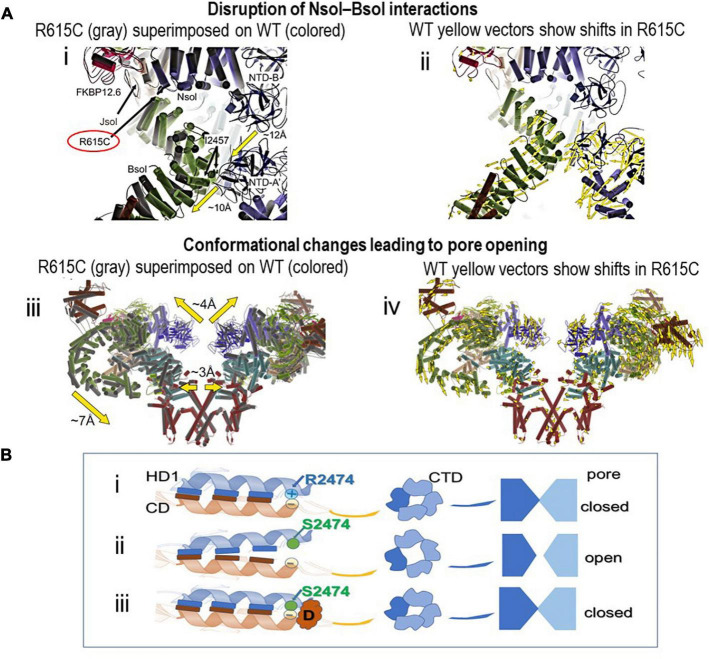
Structural changes associated with RyR mutations. **(A)** CryoEM images showing that the R615C mutation induces RyR1 channel opening by disrupting interactions between the Nsol and Bsol regions. **(i,ii)** show local changes around R615 as a result of the mutation, while **(iii,iv)** illustrate the resulting global changes in the protein. Alpha helices are represented as cylinders. **(i)** WT (colored) and R615C (gray) structures are superimposed, based on the Nsol region. The location of residue 615 is shown at the junction between the three solenoid regions, the N-terminal solenoid (Nsol, residues 395–630, containing Arg615), junctional solenoid (JSol, residues 1657–2145) and bridging solenoid (Bsol, residues 2146–3613). NTD-A and NTD-B are N-terminal disease hot spot regions. The lower heavy yellow arrow indicates the 10 Å movement of Bsol in the vicinity of residue 2457 resulting from the R615C mutation. This region of Bsol contacts the Nterminal domain A (NTD-A’) of a neighboring subunit. Therefore, as a result of the relative movement of Bsol, the hotspot regions NTD-A and NTD-B no longer form intersubunit interactions, the gap between the N-terminal domains of two neighboring subunits increases and interactions with the N-terminal domains of neighboring subunits are lost, resulting in substantial overall changes in RyR1 structure. **(ii)** the WT structure only is shown in the same orientation as in **(i)**. The yellow vectors show shifts >2 Å in the Bsol helices in R615C relative to WT RyR1. **(iii)** View of two of the four RyR1 subunits extending from the extreme cytoplasmic to luminal surfaces as in Figure 1A. WT (colored) and R615C (gray) RyR1 structures are superimposed. Global domain movements are indicated by heavy arrows. **(iv)** WT structure only in the same orientation as in (**iii**). Yellow vectors show shifts >2 Å throughout the protein in R615C relative to WT RyR1. FKBP12.6 shown in all panels was used as a bait for protein purification. The Figure has been modified from Figure 3 (Woll et al., 2021) under a Creative Commons copyright license CC BY 4.0. Labeling only has been modified. **(B)**. A cartoon of RyR2 illustrating hypothetical downstream effects of partial unzipping of an interaction between helices in HD1 (in blue) and in the central domain (CD in orange) due to the gain-of-function R2474S mutation (Kobayashi et al., 2010). The mutation likely interrupts an ionic interaction between R2474 and a negatively charged residue in a CD helix. **(i)**. Interactions between these helices in the WT protein potentially maintain the C-terminal domain (CTD) ring around the pore entrance and the pore itself in the closed conformation. The interacting helices in one subunit only are shown. The residues of interest are shown as circles, the elongated bars indicate unspecified interactions between other residues in the helices that help maintain the interdomain interaction. Inter-subunit interactions between the four CTDs are shown, while only two of the four pore subunits are shown for simplicity. **(ii)**. The R2474S substitution abolishes the ionic interaction and leads to partial unzipping of the HD1/CD interdomain interaction. This alters the inter-subunit association between the four CTDs, expanding the CTD ring and opening the pore. **(iii)**. Introduction of a drug (D) that stabilizes the interdomain interaction in the mutant protein restores the normal zipping and returns the CTD ring and the pore to the closed conformation.

Ideally, for high resolution analysis of the effect of a mutation, the structures of both wild type and mutant RyR2 should be solved using identical preparation procedures and structural data should be correlated with single channel data of proteins from the same source. The preferable source of RyR2 protein is mammalian muscle where post translational modifications are closest to those in humans, however, this generally requires the development of animal models to source sufficient protein. The process is painstakingly slow and only one study thusfar with pig RyR1 containing the malignant hyperthermia mutation, R615C, ticks most of these boxes (Woll et al., 2021). In this study, RyR1 channels from R615C pig muscle show the classic gain-of-function phenotype (Fill et al., 1990; MacLennan and Chen, 1993). Arg615 is located at a critical interface between three solenoid regions (Figure 2). Interactions between Arg615 and Asn1678 in Jsol or Glu2175 in Bsol are lost in R615C so that Bsol and Jsol pivot by ∼2–3 Å relative to Nsol. Notably, the small rotation at the mutation site is amplified in Bsol to an ∼10 Å rotation relative to the N-terminal domain of the neighboring subunit (Figure 2A,i,ii). Overall these changes result in the cytosolic cap expanding and its corners move down toward the SR membrane (Figure 2A,iii,iv). The movement of Jsol results in expansion of the C-terminal region and S6 helix to increase pore diameter and allow Ca^2+^ flow. The mutation reduces the ability of the channel to close, inducing partial channel opening with a defined pathological conformation. The results reveal a chain of specific allosteric changes that alter channel gating in response to a mutation >130 Å from the pore.

Different terminologies are applied to similar regions in both RyR1 and RyR2 throughout the high resolution CryoEM literature and in Figures 1, 2. Very generally to help with orientation in both structures, Nsol overlaps the N-terminal domain and SPRY1, Jsol overlaps SPRY1 and Helical Domain 1 and Bsol overlaps Helical Domain 1 and the Central Domain.

The investment of time and resources in developing appropriate animal models, in single channel analysis, and in solving CryoEM structures for every CPVT mutation is daunting but can be circumvented by alternative strategies to yield meaningful results. For example the RyR1 R615C and the CPVT RyR2 R176Q mutations were compared using recombinant RyRs expressed in HEK293 cells, with phenotype assessed with Ca^2+^ transients in those cells and [^3^H]ryanodine binding to RyR2 (Iyer et al., 2020). Structural changes in RyR1 R615C, assessed by comparison with their previous WT RyR1 structure (Steele and Samso, 2019), were similar to those in pig RyR1 R615C (Woll et al., 2021). Structurally, RyR2 R176 and RyR1 R615 are in an equivalent location, but the functional changes were substantially weaker with RyR2 R176Q than RyR1 R615C (Iyer et al., 2020). Consistently WT and R176C RyR2 structures, determined under the same conditions as RyR1 R615C, showed negligible structural change due to the R176Q mutation. The authors conclude that the effects of neutralizing a similarly located arginine residue depended on the nature of the mutant residue and on isoform-specific properties of RyR1 and RyR2.

Structural changes can also be predicted from existing high resolution structural data. For example, the structural basis for functional changes with RyR2 CPVT C-terminal mutations expressed in HEK293 cells (Guo et al., 2021). The G4955E mutation increased both Ca^2+^-independent basal RyR2 activity and Ca^2+^-dependent increases in activity, assessed with [^3^H]ryanodine binding and single channel currents. The predictions suggest that the introduced negative charge disrupted C-terminal interfaces between neighboring subunits to destabilize channel closure. Conversely G4955K introduced a positive charge and suppressed channel activity by strengthening the interface interactions. Other mutations P4902S and E4950K increased Ca^2+^ activation but, as with RyR2 R176Q (Iyer et al., 2020), had no effect on basal RyR2 activity. Four mutations (P4902S, P4902L E4950K, G4955E) increased caffeine-induced Ca^2+^ release and reduced the threshold for SOICR (Guo et al., 2021). The Ca^2+^ activation site (3667–4253), caffeine-activation site (around W4645) and luminal Ca^2+^ sensor (around E4872) are in the vicinity of the four mutation sites and close to the S6/C-terminal domain junction (Zhang et al., 2014; Murayama et al., 2018). Allosteric interaction predictions have been used to show that cytoplasmic Ca^2+^ and caffeine binding is communicated to the channel gate *via* a cascade of overlapping interactions that control RyR1 activity (Chirasani et al., 2021) and are likely to similarly influence RyR2 activity.

The examples above show that functional Ca^2+^ release and [^3^H]ryanodine binding, as well as structural information about individual RyR2 mutations can be obtained with recombinant proteins. However, contractile function obviously cannot be assessed in HEK293 cells. An alternative in patient-specific pluripotent stem cell-derived cardiomyocytes (hiPSC-CMs), provided a contractile expression system to demonstrate both contractile and electrical abnormalities with SR Ca^2+^ leak for a CPVT patient with the RyR2 D3638A mutation (Acimovic et al., 2018). *In silico* analysis of the mutation site based on WT RyR2 structure (Peng et al., 2016), revealed that Asp3638 is located near the Ca^2+^ activation site in a conserved central region, at a contact between alpha helices with positively charged residues on one side and negatively charged residues on the other. Removing Asp3638 weakened the interactions, reducing the ability of the central domain to communicate conformational changes to the pore.

It is not possible at present to accurately predict the functional consequence of CPVT mutations based on their location because the functional significance of most intra-domain, inter-domain and inter-subunit interactions are unknown. However, it is worth noting that the effects of the mutations are slowly providing information on the function of some of these interactions within the protein.

## Approaches to Reducing Ca^2+^ Leak Through RyR2

There are excellent detailed reviews of pathological RyR2 Ca^2+^ leak and current treatments for cardiac arrhythmia and heart failure, e.g., (Connell et al., 2020; Priori et al., 2021). A brief summary of three particular pharmacological approaches is given in the structural context of this article. It is notable that the drugs in current clinical use to reduce Ca^2+^ leak have been developed for other purposes and found serendipitously to be effective with CPVT, rather than being rationally designed for specific CPVT mutations.

### Flecainide

Flecainide is a class IC Na^+^ channel and RyR2 blocking drug with antiarrhythmic actions and is used clinically to treat atrial fibrillation, supraventricular tachycardia and CPVT. The antiarrhythmic actions of the drug are complex and include its block of both Na^+^ and RyR2 channels. Multiple anti-arrhythmic actions of flecainide are proposed (Steele et al., 2013; Salvage et al., 2018; Connell et al., 2020; Bannister et al., 2021; Priori et al., 2021). The primary mechanisms of flecainide’s efficacy with CPVT are strongly debated and all mechanisms may contribute to varying degrees depending the experimental protocol, and whether atrial or ventricular myocytes are examined. The action of flecainide in blocking current flow from cytoplasm to lumen though the pore has been extensively studied and likely reduces Ca^2+^ release by blocking counterion flow (Bannister et al., 2021). This blocking action of flecainide would presumably reduce current flowing through RyR2 during systole and diastole and be independent of the location of the mutation. Flecainide can also activate RyR2 channels at low concentrations, or inhibit at higher concentrations, independently of the direction of current flow (Salvage et al., 2021; Dulhunty et al., 2022). This indicates binding to RyR2 at cytoplasmic sites distant from the pore, however, the location of these sites is as yet unknown. The activation may contribute to occasional paradoxical pro-arrhythmic effects of the drug.

### Dantrolene

Dantrolene is used clinically to treat Ca^2+^ leak through RyR1 with RyR1 N-terminal mutations like RyR1 R615C. It is effective in treating CPVT in animal models of heart failure and CPVT (Kobayashi et al., 2010; Walweel et al., 2017). Dantrolene decreases the number of premature contractions in CPVT patients, in a mutation-dependent manner, more effectively with N-terminal and central domain mutations than with mutations outside these regions and is essentially ineffective with transmembrane domain mutations (Penttinen et al., 2015). Dantrolene is thought to act by reinforcing the action of calmodulin (CaM) in stabilizing the interaction between the N-terminus and central domains of both RyR1 and RyR2, as suggested by the unzipping model of arrhythmogenesis in CPVT (Figure 2B). Dantrolene provides an example of a very broad domain-specific action.

### JTV519 and S107

Rycal compounds including benzothiazepine derivatives JTV519 and S107, belong to the same class of drug as Diltiazem, a voltage-gated Ca^2+^ channel blocker. Both JTV519 (also known as K201) and S107 reduce Ca^2+^ leak by stabilizing FKBP12.6 binding to RyR2. As with dantrolene, JTV519 is more effective with N-terminal and central domain RyR2 mutations than with C-terminal domain mutations (Tateishi et al., 2009), again showing a broad domain-specific action and providing a further example of the unzipping model in CPVT (Figure 2B). JTV519 has off-target actions that can be pro-arrhythmic, thus reducing its clinical usefulness (Connell et al., 2020). S107 is more specific for RyR2 and FKBP12.6 binding, reducing Ca^2+^ leak and preventing arrhythmia in a mouse model of CPVT (Lehnart et al., 2008) and decreasing DADs in hiPSC-derived cardiomyocytes (Sasaki et al., 2016). As of 2020, there were no clinical trial results for S107 (Connell et al., 2020). A related compound S48168 is effective in improving muscle function in a mouse model of Duchenne muscular dystrophy (Capogrosso et al., 2018) and is undergoing clinical trials (^1^ARDSLEY, N.Y., Dec. 17, 2019/PRNewswire/– ARMGO Pharma, Inc).

### Other Therapeutic Candidates for Catecholaminergic Polymorphic Ventricular Tachycardia

Derivatives of other therapeutic drugs are currently being investigated for RyR2 specificity and CPVT efficacy, but are not yet in clinical trials (Connell et al., 2020). Gene therapy possibly has the best potential for patient specific therapy. Several genetic techniques have been successfully tested in animal CPVT models (Bongianino et al., 2017; Pan et al., 2018; Priori et al., 2021).

## Potential for Personalized Medicine

### Mutation-Specific Drugs

Notably, all drugs thusfar in use or in development to treat CPVT, globally target Ca^2+^ release from RyR rather changes due to the specific CPVT mutation. As a result, essential RyR2 modulation by endogenous factors during systole/diastole may also be compromised. The ideal personalized drug would target specific local conformational changes produced by the mutation. However, the potential for design of specific drugs for individual mutations is limited, given the number of RyR2 CPVT mutations so far identified and still emerging. A less ideal but feasible option may be the design drugs for groups of mutations in specific domains. As mentioned above, both dantrolene and JTV519 are more effective with N-terminal and central domain mutations than with transmembrane or C-terminal mutations. A compound/peptide/drug that stabilized and/or destabilized specific interfaces that convey conformational changes from a particular cytoplasmic domain to the central and C-terminal domains would be appropriate for a group of mutations that impact on the strength of a common interface interaction.

In conclusion, the potential for personalized medicine to treat RyR2 CPVT is rapidly increasing as a result of increased availability of genetic testing to identify the mutation, advances in cryoEM structural analysis technology and the development of gene therapy techniques.

## Data Availability Statement

Publicly available datasets were analyzed in materials cited in this manuscript.

## Author Contributions

AD was responsible for writing this manuscript.

## Conflict of Interest

The author declares that the research was conducted in the absence of any commercial or financial relationships that could be construed as a potential conflict of interest.

## Publisher’s Note

All claims expressed in this article are solely those of the authors and do not necessarily represent those of their affiliated organizations, or those of the publisher, the editors and the reviewers. Any product that may be evaluated in this article, or claim that may be made by its manufacturer, is not guaranteed or endorsed by the publisher.

## Footnotes

1
https://www.prnewswire.com/news-releases/armgo-pharma-inc-announces-clinical-trial-of-arm210s48168-for-the-treatment-of-ryanodine-receptor-type-1-related-myopathies-300975427.html
